# Mixed occupancy: the crystal structure of scheelite-type LiLu[MoO_4_]_2_

**DOI:** 10.1107/S2056989024004365

**Published:** 2024-05-17

**Authors:** Ingo Hartenbach, Robin F. Hertweck

**Affiliations:** a University of Stuttgart, Institute of Inorganic Chemistry, Pfaffenwaldring 55, 70569 Stuttgart, Germany; b Gymnasium in der Glemsaue, Gröninger Str. 29, 71254 Ditzingen, Germany; Vienna University of Technology, Austria

**Keywords:** crystal structure, scheelite, molybdates, lithium, lutetium, mixed occupancy

## Abstract

Coarse colorless single crystals of lithium lutetium bis­[orthomolybdate(VI)], LiLu[MoO_4_]_2_, were obtained as a by-product from a reaction aimed at lithium derivatives of lutetium molybdate. The title compound crystallizes in the scheelite structure type (tetra­gonal, space group *I*4_1_/*a*) with two formula units per unit cell. The Wyckoff position 4*b* (site symmetry  ) comprises a mixed occupancy of Li^+^ and Lu^3+^ cations in a 1:1 ratio. In comparison with a previous powder X-ray study [Cheng *et al.* (2015 ▸). *Dalton Trans.* **44**, 18078–18089.] all atoms were refined with anisotropic displacement parameters.

## Abstract 2

The crystal structure of LiLu[MoO_4_]_2_, which emerged as a by-product of the synthesis to obtain lithium derivatives of lutetium molybdate, adopts the scheelite type.

## 1. Chemical context

The mineral powellite (CaMoO_4_) is one of the main sources for molybdenum on this planet. Its tetra­gonal crystal structure can be described as isotypical with that of the mineral scheelite (CaWO_4_) in space group type *I*4_1_/*a* with the *c* axis roughly twice as long as the respective *a* axis (Dickinson, 1920 ▸). The predomination of divalent cations, such as alkaline earth metals, can be changed by introducing a mixed occupancy of monovalent (*i.e.* alkali metals) and trivalent cations (*i.e.* rare-earth metals) at the respective Wyckoff position. Since the coordination number of eight around the alkaline earth metal cations in the scheelite structure usually requires larger cations, it is remarkable that the title compound also adopts the scheelite structure type although it comprises the smallest cations of both the alkali metals and the lanthanides.

## 2. Structural commentary

**Figure 1:**
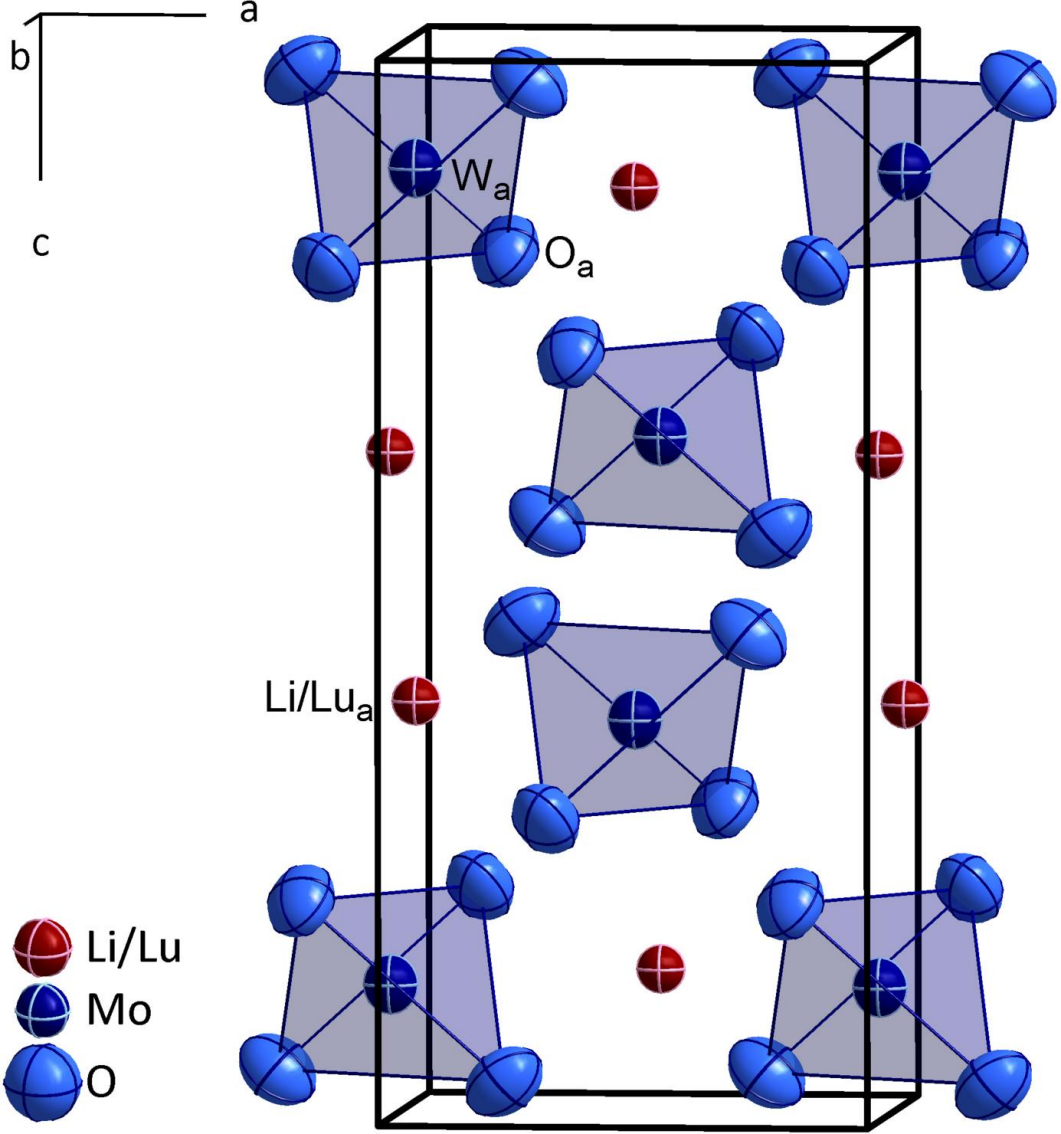
The augmented unit cell of LiLu[MoO_4_]_2_ in a view approximately along [010], with the [MoO_4_]^2–^ anions in polyhedral representation and displacement ellipsoids drawn at the 95% probability level. Atomic positions marked with the subscript "a" build up the asymmetric unit.

**Figure 2:**
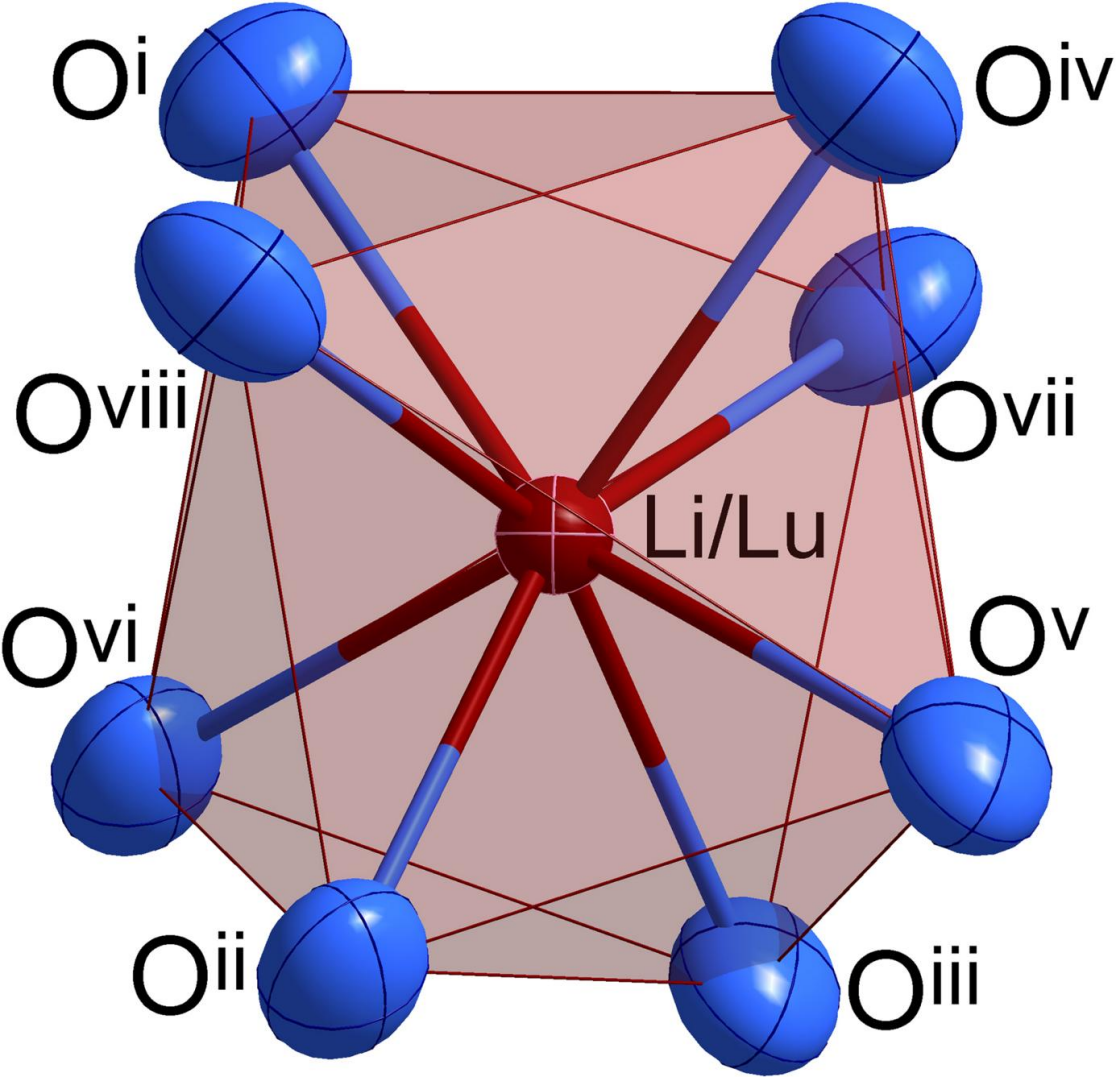
Oxidic coordination environment around the mixed cationic Li^+^/Lu^3+^ position in the shape of a trigonal dodeca­hedron; displacement ellipsoids are drawn at the 95% probability level [Symmetry codes: (i) *y* − 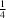 , −*x* + 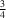 , *z* + 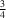 ; (ii) *x* − 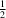 , *y*, −*z* + 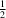 ; (iii) −*x* + 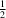 , −*y* + 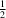 , −*z* + 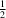 ; (iv) −*y* + 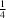 , *x* − 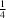 , *z* + 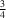 ; (v) *x* − 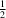 , *y* − 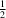 , *z* + 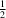 ; (vi) −*x* + 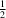 , −*y* + 1, *z* + 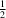 ; (vii) −*y* + 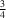 , *x* − 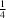 , −*z* + 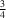 ; (viii) *y* − 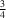 , −*x* + 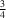 , −*z* + 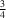 ].

In the crystal structure of LiLu[MoO_4_]_2_ (Fig. 1 ▸) the Li^+^ and Lu^3+^ cations reside at Wyckoff position 4*b* (site symmetry 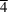 ) exhibiting a 1:1 mixed occupancy. The coordination environment around this position is built up by eight oxide anions [*d*_Li/Lu—O_ = 4 × 2.369 (3) and 4 × 2.371 (3) Å] in the shape of a trigonal dodeca­hedron (Fig. 2 ▸). The Mo^6+^ cations are situated in the centers of oxygen tetra­hedra at Wyckoff position 4*a* (site symmetry 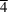 ) with distances of 4 × 1.774 (3) Å. The existence of LiLu[MoO_4_]_2_ was first mentioned by Cheng *et al.* (2015 ▸), with the crystal structure being refined by the Rietveld method on basis of X-ray data from microcrystalline powder. While their refinement of the lattice parameters [*a* = 5.10332 (11), *c* = 11.0829 (3) Å] resulted in similar values as for the current single-crystal study (see Table 1 ▸), no anisotropic displacement parameters of the refined atoms were given in the previous powder study. Furthermore, the structure refinement on basis of single-crystal data not only allows for a more accurate determination of the oxygen site, but also for a rather precise determination of the Li:Lu ratio found at Wyckoff position 4*b* (occupancy ratio 0.483 Li:0.517 Lu when refined freely). For electroneutrality, the site occupancies were fixed to ideal values (0.5:0.5) in the final refinement step.

Since Na^+^ and K^+^ cations are larger than Li^+^ cations and thus closer to the size of *Ln* ^3+^ cations, it is not astonishing that the crystal volumes of Na*Ln*[MoO_4_]_2_ and K*Ln*[MoO_4_]_2_ compounds are considerably larger than those of the respective Li*Ln*[MoO_4_]_2_ series. In case of the larger lanthanoids, lithium-containing scheelite-type structures according to the formula Li*Ln*[MoO_4_]_2_ with *Ln* = Ce^3+^ (Egorova *et al.*, 1982 ▸) and Nd^3+^ (Kolitsch, 2001 ▸) are known so far, while for Yb^3+^ as a representative of the smaller lanthanides, the crystal structure shows deviations from the Laue group 4/*m*, crystallizing in space group *I* 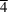 (Volkov *et al.*, 2005 ▸; Armand *et al.*, 2021 ▸). In all the aforementioned compounds, the rather small Li^+^ cations assume a mixed occupancy with the respective lanthanoid, which is also found in the crystal structures of *e.g.* Li*Ln*_5_[W_8_O_32_] for *Ln* = Y (Dorn *et al.*, 2017 ▸) and Dy–Lu (Dorn *et al.*, 2021 ▸). However, in these structures the Li^+^ cations show a sixfold coordination in contrast to the scheelite-type title compound with a coordination number of eight.

## 3. Synthesis and crystallization

Colorless single crystals of LiLu[MoO_4_]_2_, which remain stable towards atmospheric influences, were obtained as a by-product of synthesis attempts for LiLu_5_[Mo_8_O_32_]. Lithium chloride, lutetium sesquioxide and molybdenum trioxide in molar ratios of 3:8:24 were fused together in evacuated silica ampoules and treated with a stepwise temperature program with a peak value of 1123 K for four days. After a slow cooling ramp of another four days, the desired compound was obtained as a microcrystalline powder with single crystals of the title compound found in the bulk.

## 4. Refinement

Crystal data, data collection and structure refinement details are summarized in Table 1 ▸. The 1:1 ratio of Li^+^ and Lu^3+^ was reached by fixed occupation factors (0.5:0.5) of the respective atoms at Wyckoff position 4*b*.

## Supplementary Material

Crystal structure: contains datablock(s) I. DOI: 10.1107/S2056989024004365/wm5719sup1.cif

Structure factors: contains datablock(s) I. DOI: 10.1107/S2056989024004365/wm5719Isup2.hkl

CCDC reference: 2312843

Additional supporting information:  crystallographic information; 3D view; checkCIF report

## Figures and Tables

**Table 1:** Experimental details

Crystal data	
Chemical formula	LiLu[MoO_4_]_2_
*M*_r_	501.79
Crystal system, space group	Tetragonal, *I*4_1_/*a*
Temperature (K)	293
*a*, *c* (Å)	5.1052 (3), 11.0800 (7)
*V* (Å^3^)	288.78 (4)
*Z*	2
Radiation type	Ag *K*α, λ = 0.56083 Å
μ (mm^−1^)	25.07
Crystal size (mm)	0.14 × 0.09 × 0.08

Data collection	
Diffractometer	Stoe Stadivari
Absorption correction	Multi-scan [*X-RED32* (Stoe & Cie, 2019 ▸) using Gaussian integration, analogous to Coppens (1970 ▸). Afterwards scaling of reflection intensities was performed within *LANA* (Koziskova *et al.*, 2016 ▸)]
*T*_min_, *T*_max_	0.031, 0.155
No. of measured, independent and observed [*I* > 2σ(*I*)] reflections	4315, 352, 162
*R*_int_	0.037
(sin θ/λ)_max_ (Å^−1^)	0.833

Refinement	
*R*[*F* ^2^ > 2σ(*F* ^2^)], *wR*(*F* ^2^), *S*	0.019, 0.048, 0.96
No. of reflections	352
No. of parameters	15
Δρ_max_, Δρ_min_ (e Å^−3^)	1.14, −1.22

Computer programs: *X-AREA* (Stoe & Cie, 2019 ▸), *LANA* (Koziskova *et al.*, 2016 ▸), *SHELXT* (Sheldrick, 2015*a* ▸), *SHELXL* (Sheldrick, 2015*b* ▸), *DIAMOND* (Brandenburg & Putz, 2023 ▸) and *publCIF* (Westrip, 2010 ▸).

## supplementary crystallographic information

### Crystal data

**Table:**

LiLu[MoO_4_]_2_	*D*_x_ = 5.771 Mg m^−^^3^
*M**_r_* = 501.79	Ag *K*α radiation, λ = 0.56083 Å
Tetragonal, *I*4_1_/*a*	Cell parameters from 2522 reflections
*a* = 5.1052 (3) Å	θ = 3.5–31.7°
*c* = 11.0800 (7) Å	µ = 25.07 mm^−^^1^
*V* = 288.78 (4) Å^3^	*T* = 293 K
*Z* = 2	Coarse, colorless
*F*(000) = 444	0.14 × 0.09 × 0.08 mm

### Data collection

**Table:**

Stoe Stadivari diffractometer	352 independent reflections
Radiation source: Axo Ag	162 reflections with *I* > 2σ(*I*)
Graded multilayer mirror monochromator	*R*_int_ = 0.037
Detector resolution: 5.81 pixels mm^-1^	θ_max_ = 27.9°, θ_min_ = 3.5°
rotation method, ω scans	*h* = −8→8
Absorption correction: multi-scan [X-Red32 (Stoe & Cie, 2019) using Gaussian integration, analogous to Coppens (1970). Afterwards scaling of reflection intensities was performed within *LANA* (Koziskova *et al.*, 2016)]	*k* = −8→8
*T*_min_ = 0.031, *T*_max_ = 0.155	*l* = −15→18
4315 measured reflections	

### Refinement

**Table:**

Refinement on *F*^2^	0 restraints
Least-squares matrix: full	*w* = 1/[σ^2^(*F*_o_^2^) + (0.0206*P*)^2^ + 1.1482*P*] where *P* = (*F*_o_^2^ + 2*F*_c_^2^)/3
*R*[*F*^2^ > 2σ(*F*^2^)] = 0.019	(Δ/σ)_max_ < 0.001
*wR*(*F*^2^) = 0.048	Δρ_max_ = 1.14 e Å^−^^3^
*S* = 0.96	Δρ_min_ = −1.22 e Å^−^^3^
352 reflections	Extinction correction: SHELXL (Sheldrick 2015b), Fc^*^=kFc[1+0.001xFc^2^λ^3^/sin(2θ)]^-1/4^
15 parameters	Extinction coefficient: 0.044 (2)

### Special details

**Table:**

Geometry. All esds (except the esd in the dihedral angle between two l.s. planes) are estimated using the full covariance matrix. The cell esds are taken into account individually in the estimation of esds in distances, angles and torsion angles; correlations between esds in cell parameters are only used when they are defined by crystal symmetry. An approximate (isotropic) treatment of cell esds is used for estimating esds involving l.s. planes.

### Fractional atomic coordinates and isotropic or equivalent isotropic displacement parameters (Å^2^)

**Table:**

	*x*	*y*	*z*	*U*_iso_*/*U*_eq_	Occ. (<1)
Li	0.000000	0.250000	0.625000	0.00754 (18)	0.5
Lu	0.000000	0.250000	0.625000	0.00754 (18)	0.5
Mo	0.000000	0.250000	0.125000	0.01028 (19)	
O	0.2480 (4)	0.4067 (5)	0.0394 (2)	0.0175 (6)	

### Atomic displacement parameters (Å^2^)

**Table:**

	*U*^11^	*U*^22^	*U*^33^	*U*^12^	*U*^13^	*U*^23^
Li	0.0075 (2)	0.0075 (2)	0.0075 (3)	0.000	0.000	0.000
Lu	0.0075 (2)	0.0075 (2)	0.0075 (3)	0.000	0.000	0.000
Mo	0.0097 (2)	0.0097 (2)	0.0114 (3)	0.000	0.000	0.000
O	0.0203 (15)	0.0157 (14)	0.0177 (13)	−0.0012 (12)	0.0049 (11)	0.0027 (14)

### Geometric parameters (Å, º)

**Table:**

Li—O^i^	2.369 (3)	Lu—O^v^	2.371 (3)
Li—O^ii^	2.369 (3)	Lu—O^vi^	2.371 (3)
Li—O^iii^	2.369 (3)	Lu—O^vii^	2.371 (3)
Li—O^iv^	2.369 (3)	Lu—O^viii^	2.371 (3)
Li—O^v^	2.371 (3)	Lu—Lu^ix^	3.7668 (2)
Li—O^vi^	2.371 (3)	Lu—Lu^x^	3.7668 (2)
Li—O^vii^	2.371 (3)	Lu—Lu^xi^	3.7668 (2)
Li—O^viii^	2.371 (3)	Lu—Lu^xii^	3.7668 (2)
Lu—O^i^	2.369 (3)	Mo—O^xiii^	1.774 (3)
Lu—O^ii^	2.369 (3)	Mo—O^xiv^	1.774 (3)
Lu—O^iii^	2.369 (3)	Mo—O^xv^	1.774 (3)
Lu—O^iv^	2.369 (3)	Mo—O	1.774 (3)
			
O^i^—Li—O^ii^	126.20 (9)	O^iv^—Lu—O^viii^	74.75 (12)
O^i^—Li—O^iii^	126.20 (9)	O^v^—Lu—O^viii^	99.23 (6)
O^ii^—Li—O^iii^	79.57 (15)	O^vi^—Lu—O^viii^	99.23 (6)
O^i^—Li—O^iv^	79.57 (15)	O^vii^—Lu—O^viii^	132.78 (15)
O^ii^—Li—O^iv^	126.20 (9)	O^i^—Lu—Lu^ix^	158.93 (7)
O^iii^—Li—O^iv^	126.20 (9)	O^ii^—Lu—Lu^ix^	70.38 (7)
O^i^—Li—O^v^	153.18 (13)	O^iii^—Lu—Lu^ix^	37.40 (7)
O^ii^—Li—O^v^	69.36 (7)	O^iv^—Lu—Lu^ix^	101.37 (7)
O^iii^—Li—O^v^	74.75 (12)	O^v^—Lu—Lu^ix^	37.35 (7)
O^iv^—Li—O^v^	73.93 (6)	O^vi^—Lu—Lu^ix^	101.88 (8)
O^i^—Li—O^vi^	73.93 (6)	O^vii^—Lu—Lu^ix^	85.81 (7)
O^ii^—Li—O^vi^	74.75 (12)	O^viii^—Lu—Lu^ix^	131.46 (8)
O^iii^—Li—O^vi^	69.36 (7)	O^i^—Lu—Lu^x^	37.40 (7)
O^iv^—Li—O^vi^	153.18 (13)	O^ii^—Lu—Lu^x^	158.93 (7)
O^v^—Li—O^vi^	132.78 (15)	O^iii^—Lu—Lu^x^	101.37 (7)
O^i^—Li—O^vii^	74.75 (12)	O^iv^—Lu—Lu^x^	70.38 (8)
O^ii^—Li—O^vii^	153.18 (13)	O^v^—Lu—Lu^x^	131.46 (7)
O^iii^—Li—O^vii^	73.93 (6)	O^vi^—Lu—Lu^x^	85.81 (7)
O^iv^—Li—O^vii^	69.36 (7)	O^vii^—Lu—Lu^x^	37.35 (7)
O^v^—Li—O^vii^	99.23 (6)	O^viii^—Lu—Lu^x^	101.88 (8)
O^vi^—Li—O^vii^	99.23 (6)	Lu^ix^—Lu—Lu^x^	122.737 (3)
O^i^—Li—O^viii^	69.36 (7)	O^i^—Lu—Lu^xi^	70.38 (8)
O^ii^—Li—O^viii^	73.93 (6)	O^ii^—Lu—Lu^xi^	101.37 (7)
O^iii^—Li—O^viii^	153.18 (13)	O^iii^—Lu—Lu^xi^	158.93 (7)
O^iv^—Li—O^viii^	74.75 (12)	O^iv^—Lu—Lu^xi^	37.40 (7)
O^v^—Li—O^viii^	99.23 (6)	O^v^—Lu—Lu^xi^	85.81 (7)
O^vi^—Li—O^viii^	99.23 (6)	O^vi^—Lu—Lu^xi^	131.46 (7)
O^vii^—Li—O^viii^	132.78 (15)	O^vii^—Lu—Lu^xi^	101.88 (8)
O^i^—Lu—O^ii^	126.20 (9)	O^viii^—Lu—Lu^xi^	37.35 (7)
O^i^—Lu—O^iii^	126.20 (9)	Lu^ix^—Lu—Lu^xi^	122.737 (3)
O^ii^—Lu—O^iii^	79.57 (15)	Lu^x^—Lu—Lu^xi^	85.322 (6)
O^i^—Lu—O^iv^	79.57 (15)	O^i^—Lu—Lu^xii^	101.37 (7)
O^ii^—Lu—O^iv^	126.20 (9)	O^ii^—Lu—Lu^xii^	37.40 (7)
O^iii^—Lu—O^iv^	126.20 (9)	O^iii^—Lu—Lu^xii^	70.38 (7)
O^i^—Lu—O^v^	153.18 (13)	O^iv^—Lu—Lu^xii^	158.93 (7)
O^ii^—Lu—O^v^	69.36 (7)	O^v^—Lu—Lu^xii^	101.88 (8)
O^iii^—Lu—O^v^	74.75 (12)	O^vi^—Lu—Lu^xii^	37.35 (7)
O^iv^—Lu—O^v^	73.93 (6)	O^vii^—Lu—Lu^xii^	131.46 (7)
O^i^—Lu—O^vi^	73.93 (6)	O^viii^—Lu—Lu^xii^	85.81 (7)
O^ii^—Lu—O^vi^	74.75 (12)	Lu^ix^—Lu—Lu^xii^	85.322 (5)
O^iii^—Lu—O^vi^	69.36 (7)	Lu^x^—Lu—Lu^xii^	122.737 (3)
O^iv^—Lu—O^vi^	153.18 (13)	Lu^xi^—Lu—Lu^xii^	122.737 (3)
O^v^—Lu—O^vi^	132.78 (15)	O^xiii^—Mo—O^xiv^	106.65 (10)
O^i^—Lu—O^vii^	74.75 (12)	O^xiii^—Mo—O^xv^	106.65 (10)
O^ii^—Lu—O^vii^	153.18 (13)	O^xiv^—Mo—O^xv^	115.3 (2)
O^iii^—Lu—O^vii^	73.93 (6)	O^xiii^—Mo—O	115.3 (2)
O^iv^—Lu—O^vii^	69.36 (7)	O^xiv^—Mo—O	106.65 (10)
O^v^—Lu—O^vii^	99.23 (6)	O^xv^—Mo—O	106.65 (10)
O^vi^—Lu—O^vii^	99.23 (6)	Mo—O—Li^iii^	130.25 (16)
O^i^—Lu—O^viii^	69.36 (7)	Mo—O—Li^xvi^	120.42 (15)
O^ii^—Lu—O^viii^	73.93 (6)	Li^iii^—O—Li^xvi^	105.25 (12)
O^iii^—Lu—O^viii^	153.18 (13)		

Symmetry codes: (i) *y*−1/4, −*x*+3/4, *z*+3/4; (ii) *x*−1/2, *y*, −*z*+1/2; (iii) −*x*+1/2, −*y*+1/2, −*z*+1/2; (iv) −*y*+1/4, *x*−1/4, *z*+3/4; (v) *x*−1/2, *y*−1/2, *z*+1/2; (vi) −*x*+1/2, −*y*+1, *z*+1/2; (vii) −*y*+3/4, *x*−1/4, −*z*+3/4; (viii) *y*−3/4, −*x*+3/4, −*z*+3/4; (ix) −*x*, −*y*, −*z*+1; (x) −*x*+1/2, −*y*+1/2, −*z*+3/2; (xi) −*x*−1/2, −*y*+1/2, −*z*+3/2; (xii) −*x*, −*y*+1, −*z*+1; (xiii) −*x*, −*y*+1/2, *z*; (xiv) −*y*+1/4, *x*+1/4, −*z*+1/4; (xv) *y*−1/4, −*x*+1/4, −*z*+1/4; (xvi) *x*+1/2, *y*+1/2, *z*−1/2.

## References

[bb1] Armand, P., Granier, D., Reibel, C., Daenens, L. & Tillard, M. (2021). *J. Alloys Compd.* **884**, 161074.

[bb2] Brandenburg, K. & Putz, H. (2023). *DIAMOND5*. Crystal Impact GbR, Bonn, Germany.

[bb3] Cheng, F., Xia, Z., Molokeev, M. S. & Jing, X. (2015). *Dalton Trans.* **44**, 18078–18089. 10.1039/c5dt02760h 26416313

[bb4] Coppens, P. (1970). *The Evaluation of Absorption and Extinction in Single-Crystal Structure Analysis*. In *Crystallographic Computing*, edited by F. R. Ahmed, pp. 255–270. Copenhagen: Munksgaard.

[bb5] Dickinson, R. G. (1920). *J. Am. Chem. Soc.* **42**, 85–93.

[bb6] Dorn, K. V., Blaschkowski, B., Bamberger, H., van Slageren, J., Widenmeyer, M., Weidenkaff, A., Suard, E. & Hartenbach, I. (2021). *J. Alloys Compd.* **868**, 159147.

[bb7] Dorn, K. V., Schustereit, T., Strobel, S. & Hartenbach, I. (2017). *Z. Anorg. Allg. Chem.* **643**, 2050–2056.

[bb8] Egorova, A. N., Maier, A. A., Nevskii, N. N. & Provotorov, M. V. (1982). *Neorg. Mater.* **18**, 2036–2038.

[bb9] Kolitsch, U. (2001). *Z. Kristallogr. Cryst. Mater.* **216**, 449–454.

[bb10] Koziskova, J., Hahn, F., Richter, J. & Kožíšek, J. (2016). *Acta Chim. Slovaca*, **9**, 136–140.

[bb11] Sheldrick, G. M. (2015*a*). *Acta Cryst.* A**71**, 3–8.

[bb12] Sheldrick, G. M. (2015*b*). *Acta Cryst.* C**71**, 3–8.

[bb13] Stoe & Cie (2019). *X-RED32* and *X-AREA.* Stoe & Cie, Darmstadt, Germany.

[bb14] Volkov, V., Cascales, C., Kling, A. & Zaldo, C. (2005). *Chem. Mater.* **17**, 291–300.

[bb15] Westrip, S. P. (2010). *J. Appl. Cryst.* **43**, 920–925.

